# A fusion protein’s weak link: functional constraints revealed by inhibitory peptide interaction with the parainfluenza fusion protein

**DOI:** 10.1128/mbio.02741-25

**Published:** 2026-04-14

**Authors:** Maximilian Crosby, Ariel J. Kuhn, Gillian Zipursky, Tara C. Marcink, Kyle Stearns, Elizabeth B. Sobolik, Dariia Vyshenska, Jee Ching Mook, Alexander L. Greninger, Matteo Porotto, Samuel H. Gellman, Anne Moscona

**Affiliations:** 1 Center for Host–Pathogen Interaction, Columbia University 21611, New York, New York, USA; 2 Department of Pediatrics, Columbia University 21611, New York, New York, USA; 3 Department of Chemistry, University of Wisconsin 201643, Madison, Wisconsin, USA; 4 Department of Laboratory Medicine and Pathology, University of Washington 7284, Seattle, Washington, USA; 5 Department of Experimental Medicine, University of Campania “Luigi Vanvitelli” 18994, Caserta, Italy; 6 Department of Microbiology and Immunology, Columbia University 21611, New York, New York, USA; 7 Department of Physiology and Cellular Biophysics, Columbia University 21611, New York, New York, USA; Washington University in St. Louis, St. Louis, Missouri, USA

**Keywords:** viral entry, parainfluenza virus, antiviral agents, membrane fusion, protease resistance

## Abstract

Human parainfluenza viruses (HPIVs) cause significant respiratory illnesses including croup and pneumonia. HPIV infection begins with fusion of viral and host cell membranes, driven by the coordinated actions of the attachment (HN) and fusion (F) glycoproteins, which together form the fusion/entry complex. We have described fusion inhibitory peptides that are derived from the heptad repeat (HRC) domain of the F glycoprotein and have potent antiviral activity against HPIV3 and other paramyxoviruses. These peptides inhibit fusion by binding to the transiently exposed N-terminal heptad repeat (HRN) segments of the F prehairpin intermediate and preventing the six-helix bundle (6HB) formation that is required for the membrane fusion process. We report viral variants that escape inhibition by an HRC-derived α/β-peptide of HPIV3 through an HN mutation that enhances HN’s activation of F and a mutation in F’s HRN domain that destabilizes the 6HB. The F HRN domain bearing the alteration shows reduced α-helicity relative to the wild-type HRN and forms an assembly with the HRC domain that is destabilized relative to the wild-type 6HB. This viral variant is not resistant to an HRC-derived α-peptide inhibitor that forms a more stable 6HB relative to the HRC-derived α/β-peptide. The emergence of this variant suggests that improved inhibitor potency against HPIV3 could be achieved by increasing the stability of the α/β-peptide/HRN 6HB. The unique mutation in F that reduces sensitivity to inhibitor also compromises viral fitness in a human airway model and impairs viral infection, presumably as a result of diminished post-fusion state stability.

**IMPORTANCE:**

Human parainfluenza viruses (HPIVs) are major causes of lower respiratory tract disease, including croup and pneumonia. Viral entry is initiated when the viral receptor binding protein engages its host receptor and activates the viral fusion (F) protein. The F protein then undergoes an essential refolding process, inserting into the host membrane and collapsing into a stable six-helix bundle (6HB) structure to drive membrane fusion. This step is a promising target for antiviral peptides, which can block infection by preventing the formation of the 6HB structure. Here, we examined an HPIV variant that emerged under selection pressure from such a prototype antiviral peptide. A single mutation in F destabilized the 6HB and disrupted refolding, enabling viral spread in the presence of the antiviral peptide. However, this adaptation within a highly conserved region imposed a substantial fitness cost in the airway, underscoring the critical constraints on fusion protein function during infection *in vivo*.

## INTRODUCTION

Human parainfluenza viruses (HPIVs) are paramyxoviruses that cause a wide range of respiratory illnesses, including croup, bronchitis, bronchiolitis, and pneumonia. These viruses account for 30%–40% of childhood croup cases, with infants, children, and immunocompromised individuals being particularly vulnerable (1–5). Currently, there are no Food and Drug Administration-approved vaccines or antiviral treatments available for any HPIV serotype, and the eventual development of a vaccine is unlikely to fully eliminate HPIV-related disease. HPIV3 is responsible for more hospitalizations than HPIV1, HPIV2, or HPIV4 (2, 6, 7). Corticosteroids have reduced hospitalizations for HPIV1-associated respiratory disease (8); however, there are no available treatment options for infections caused by HPIV2, HPIV3, or HPIV4 (9–11).

As is the case for other paramyxoviruses, HPIV3 infection begins with the fusion of viral and host cell membranes, driven by the coordinated actions of attachment glycoproteins (HN for HPIV3; H or G for related paramyxoviruses) and fusion (F) glycoproteins, which together form the fusion complex required for viral entry (12–18). HPIV3 HN binds to a host cell surface receptor, triggering the activation of the F protein. The homotrimeric F protein is initially anchored to the viral envelope in a metastable prefusion state by its transmembrane segments and adjacent C-terminal heptad repeat (HRC) domains and undergoes structural transitions upon HN-receptor engagement. The N-terminal heptad repeat (HRN) domains of F extend, inserting the fusion peptide segments into the host cell membrane and forming a transient prehairpin intermediate. This is followed by refolding of the F protein, resulting in the assembly of a stable six-helix bundle (6HB), a step tightly coupled to the membrane fusion process (12, 13, 19–23).

Peptides corresponding to the HRC domain can inhibit fusion by binding to the transiently exposed HRN segments of the short-lived prehairpin intermediate and preventing 6HB formation (24–26). The HRN domain of F is highly conserved structurally across paramyxoviruses, and HRC peptide inhibitors are effective for this large group of viruses including Nipah and measles viruses (24, 27–33). We have previously described fusion inhibitory peptides derived from the HPIV3 F HRC domain that display potent antiviral activity against HPIV3 and other paramyxoviruses (24, 27–31, 34–38). To enhance antiviral activity of HPIV3-derived HRC peptides *in vivo*, we have used two complementary strategies, lipid conjugation (27, 28, 37, 39) and β-amino acid incorporation (33, 40–42) to improve the peptides’ resistance to degradation by proteases. Attaching a lipophilic unit to peptides composed entirely of α-amino acid residues (α-peptides) improves pharmacokinetic properties, decreases protease sensitivity, and increases antiviral potency by several logs relative to unmodified α-peptides (27, 28, 37, 39). We have shown that replacing specific α-amino acid residues with β-amino acid residues to generate an “α/β-peptide” can significantly reduce susceptibility to proteolysis (33, 40–42). In recent work, we generated a prototype peptide combining lipid conjugation and α-to-β substitution that showed decreased susceptibility to proteolytic degradation, enhanced pharmacokinetic properties, and potent anti-HPIV3 activity *in vivo* (33). To arrive at our current prototype antiviral peptide, we started with an α-peptide based on the HPIV3 F HRC domain that contains two substitutions, E459V (27, 35) and A463I (27), designated “α-VI.” We then tested a series of α/β-peptide derivatives to arrive at the candidate lipopeptide “α/β-VI-8-PEG4-Chol.” α/β-VI-8 co-assembled with HPIV3 HRN to form a 6HB that was structurally similar to that formed by α-VI and HPIV3 HRN, as established by x-ray crystallography. The α/β-VI-8 + HRN 6HB was less stable than the α-VI + HRN 6HB, as indicated by variable-temperature circular dichroism (CD) measurements. However, α/β-VI-8-PEG4-Chol was more potent against HPIV3 *in vivo* than the corresponding all-α lipopeptide, α-VI-PEG4-Chol (33). These findings highlighted how increasing resistance to proteolysis via α-to-β modification improves efficacy *in vivo*. These data also suggested that further improvement in *in vivo* potency vs HPIV3 could be achieved by increasing the stability of the α/β-peptide + HRN 6HB (37, 43).

Here, we asked whether the reduced stability of the 6HB assembly formed by α/β-VI-8 might allow for emergence of peptide-resistant variant viruses. Selecting viruses in the presence of the prototype α/β-substituted HRC peptide resulted in resistant viral variants that escape the α/β-VI-8-PEG4-Chol peptide’s inhibitory effect but remain sensitive to α-VI-PEG4-Chol. The variants bear an alteration in HN that enhances HN’s activation of F’s conformational transition, showing that alterations in HN that enhance its triggering function can contribute to resistance to fusion inhibitors. The variants also bear an alteration in F’s HRN domain that destabilizes the 6HB and confers fusion inhibitor resistance. Surprisingly, this mutation handicaps viral entry and renders the variants dependent on the fusion inhibitory peptide for viral entry. When α/β-VI-8-PEG4-Chol peptide is present to “repair” the domain’s destabilized structure, the F glycoprotein can more effectively mediate membrane merger.

## RESULTS

### Viral evolution under the selective pressure of α/β-VI-8-PEG4-Chol

**Fig 1:**
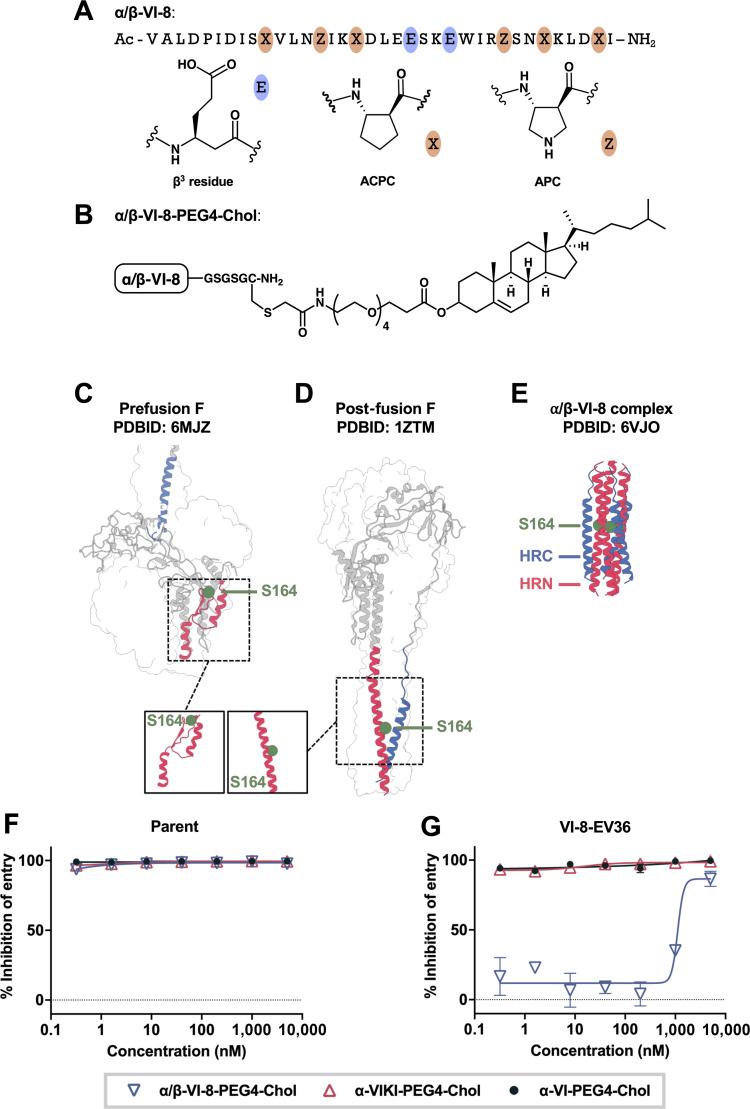
Resistance to inhibition by α/β-VI-8-PEG4-Chol. (**A**) Sequence of α/β-VI-8. β^3^ residues (blue ovals) and cyclically constrained β residues (red ovals) are highlighted within the α/β-VI-8 sequence. (**B**) Structure of PEG4 cholesterol conjugate affixed to α/β-VI-8. (**C–E**) Atomic models of (**C**) prefusion F, (**D**) post-fusion F, and (**E**) α/β-VI-8 six-helix bundle complex with S164 highlighted (green spheres). (**F and G**) Inhibition of viral entry for parent (**F**) or α/β-VI-8-resistant virus (**G**) with serial dilutions of either α/β-VI-8-PEG4-Chol (blue triangles), α-VIKI-PEG4-Chol (red triangles), or α-VI-PEG4-Chol (dark blue dots). Percent inhibition was quantitated by plaque counting normalized to no treatment.

Viral evolution experiments were conducted by using either a potent all-α broad-spectrum HRC inhibitory peptide we have identified, α-VIKI-PEG4-Chol (27), or α/β-VI-8-PEG4-Chol (Fig. 1A and B) (33) to select for variants that are infectious in the presence of the peptide inhibitor. The parental virus in this experiment is a recombinant virus that represents a field strain with an HN H552Q mutation to permit assessment of fusion in cultured cells (CI-1-HN H552Q) (16, 44–46). During selection with α/β-VI-8-PEG4-Chol, we detected two escape variant mutations in the same viral isolate: one at 100% allele frequency in HN (N556D) that we have identified previously in laboratory-adapted strains of HPIV3 (47) and one at 36% allele frequency in the HRN domain of F (S164P). This variant virus will be referred to as VI-8-EV36, for “α/β-VI-8-PEG4-Chol escape variant with 36% allele frequency of S164P” (see National Center for Biotechnology Information [NCBI] BioProject PRJNA1302008). During selection with α-VIKI-PEG4-Chol under the same conditions, no resistant variant viruses emerged. The α/β-VI-8-resistance mutation S164P in the pre-fusion F (Fig. 1C) occurs in an unfolded loop (amino acids 160–169) that undergoes significant rearrangement to become α-helical in the post-fusion F (Fig. 1D). This rearrangement is driven by the co-assembly of the HRN and HRC domains to form the 6HB. We previously solved the structure of the 6HB assembly formed by HPIV3 HRN and α/β-VI-8 (33), and the site of the α/β-VI-8-resistance mutation S164P is shown in the context of this 6HB complex, which contains the native serine at the indicated position (Fig. 1E). While α/β-VI-8-PEG4-Chol was effective in inhibiting entry for the parental virus, entry by the escape variant, as measured by plaque formation, was resistant to α/β-VI-8-PEG4-Chol at every concentration tested (Fig. 1F and G). The acquired escape mutations present in VI-8-EV36 did not allow the virus to escape inhibition by α-VIKI-PEG4-Chol or α-VI-PEG4-Chol.

### The presence of the peptide inhibitor selectively enriches for the S164P mutation in F

**Fig 2:**
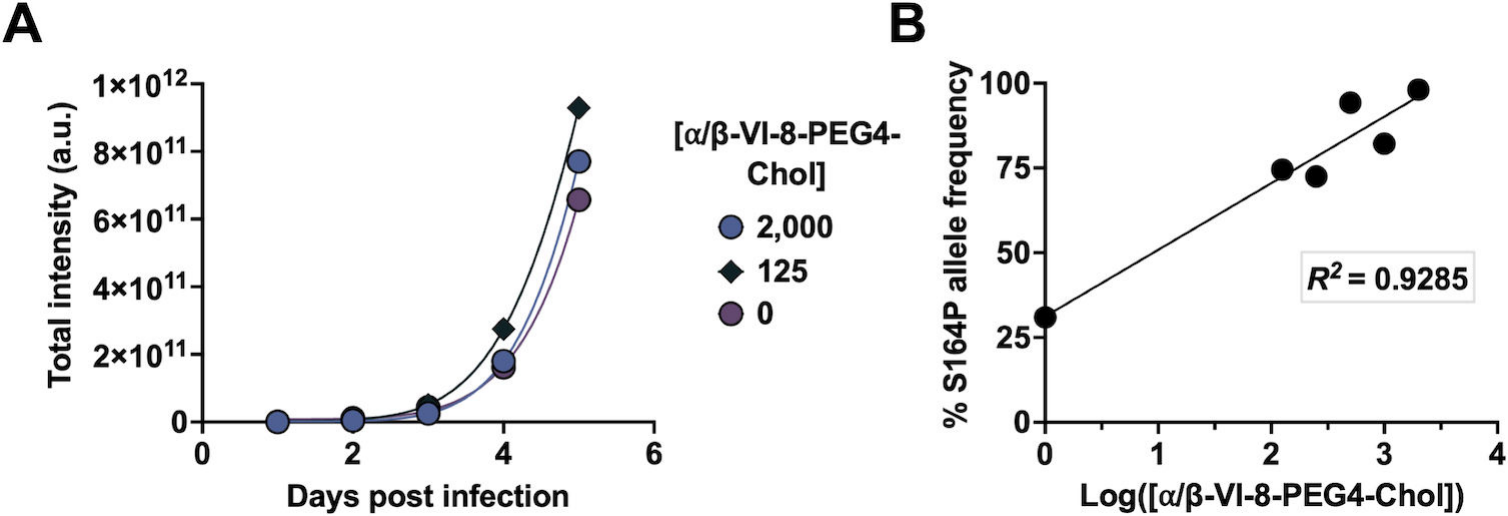
α/β-VI-8-PEG4-Chol selects for the F-S164P mutation in a dose-dependent fashion. (**A**) α/β-VI-8-resistant virus containing HN-N556D and F-S164P mutations (VI-8-EV36) was grown for 5 days in varying concentrations of α/β-VI-8-PEG4-Chol peptide. Infection was quantified by indirect immunofluorescence. (**B**) S164P allele frequency was measured at 6 days post-infection for all viruses shown in panel** A**. The coefficient of determination *R*^2^ is shown as an indicator of goodness of fit for the linear regression. Concentrations are in nM for panels **A** and **B**.

To determine whether the F S164P mutation is advantageous or deleterious in the presence of inhibitor during multi-cycle spread in culture, VI-8-EV36 was grown for 5 days in monolayer cultures in the presence of 0 to 2,000 nM α/β-VI-8-PEG4-Chol peptide. Throughout the incubation period, the cells were overlayed with carboxymethylcellulose to ensure that all viral spread was cell to cell. Viral spread continued regardless of the presence of this peptide (Fig. 2A). The presence of higher concentration of peptide during viral growth in culture led to an increase in allele frequency of the F S164P mutation, to 82% allele frequency at 6 days post-infection (dpi) after removal of the overlay and collection of virus (Fig. 2B; referred to subsequently as “VI-8-EV82”). During the growth of this virus in monolayer culture without overlay in the absence of peptide, the allele frequency of the F-S164P mutation fell to 10% at 3 dpi (Fig. S1A). An F S164P bearing virus with F S164P at 95% allele frequency (“VI-8-EV95”) and parental virus were titered by plaque assay, and genome copy number was determined. The F S164P variant has a reduced ratio of infectious particles (plaque-forming unit [PFU]) to genome copies (Fig. S1B), indicating the presence of more non-infectious particles in the VI-8-EV95 and the decreased fitness of this viral population with respect to the parent virus. Figure S1B also highlights the resistance of VI-8-EV95 to α/β-VI-8-PEG4-Chol.

### The S164P mutation in F destabilizes the F HRN, disrupting α-helicity

**Fig 3:**
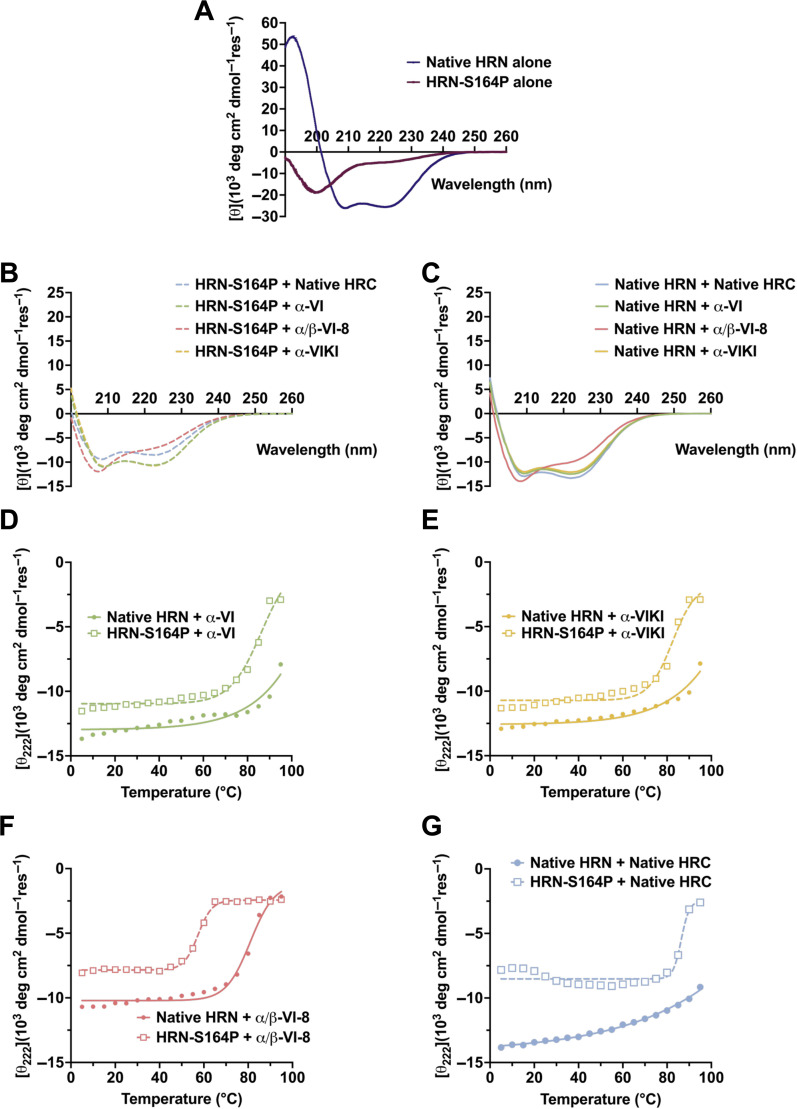
Circular dichroism analysis of HRN peptides and HRC + HRN combinations. (**A**) Full-spectrum circular dichroism scan at 25°C of individual HRN-parental or HRN-S164P peptides at 50 µM in 10 mM aqueous phosphate buffer, pH 7.4. (**B and C**) Full-spectrum circular dichroism scan at 25°C of binary mixtures of (**B**) HRN-S164P with either α-VI, α-VIKI, α/β-VI-8, or native HRC or (**C**) HRN-parental with either α-VI, α-VIKI, α/β-VI-8, or native HRC (100 µM total peptide concentration). (**D–G**) Variable-temperature circular dichroism data for binary mixtures of the HRN-parental or HRN-S16P peptide with (**D**) α-VI, (**E**) α-VIKI, (**F**) α/β-VI-8, or (**G**) native HRC (100 µM total peptide concentration).

We employed CD measurements to evaluate how the S164P modification affects the HPIV3 HRN domain in isolation and the impact on co-assembly of HRN with HRC. At 50 μM, the native HRN peptide exhibited a strong α-helical signature, with minima at 208 and 222 nm (Fig. 3A). It is likely that the peptide self-assembles under these conditions, perhaps forming a trimer analogous to the HRN trimer at the core of the 6HB. In contrast, CD data for 50 μM HRN-S164P peptide under these conditions suggested a completely unfolded peptide. Increasing the concentration of HRN-S164P to 300 μM led to the development of a weak α-helical CD signature (Fig. S2), which suggests a weak propensity of HRN-S164P for self-assembly. These data are consistent with the expectation that the S164P mutation weakens the α-helical propensity of the HRN domain.

Mixing the HRN-S164P peptide with the native HPIV3 HRC peptide (50 μM each) resulted in an α-helical CD signature of moderate intensity (Fig. 3B). Similar CD signatures were observed for mixtures of HRN-S164P with α-VI or α-VIKI. These data suggest partial formation of 6HB assemblies in each case. The CD signature for the mixture of HRN-S164P with α/β-VI-8 had a somewhat different shape, with the most pronounced minimum at 205 nm, as expected for a helical assembly containing an α/β-peptide (40). Similar CD signatures were observed for the analogous mixtures containing the native HRN peptide (Fig. 3C), although in these cases the minima were more pronounced. This observation suggests that the native HRN forms a more stable 6HB assembly with each of the four HRC peptides relative to HRN-S164P.

Further evidence that the HRN-S164P forms less stable 6HB assemblies relative to the native HRN was obtained from variable-temperature CD measurements (Fig. 3D through G). For all eight possible pairings among the two HRN peptides (native or S164P) and the four HRC peptides (native, α-VI, α-VIKI, or α/β-VI-8), mean residue ellipticity (MRE) was monitored at 222 nm, one of the minima characteristic of α-helix formation, as the sample was heated. For each pairing, MRE changed relatively little in the lower temperature range, but the value became less negative (smaller absolute value) in the high temperature range. For the pairs containing the native HRN and the native HRC, α-VI, or α-VIKI, the change in MRE was modest even at the highest temperature. This behavior suggests that the 6HB in each case is very stable, with little disruption at elevated temperature. In contrast, native HRC plus α/β-VI-8 displayed substantial changes at high temperatures, which is consistent with our previous report (33). This evidence of extensive 6HB disruption at high temperatures supports the conclusion that the 6HB formed by α/β-VI-8 is less stable than 6HBs formed by the other HRC peptides.

For each of the HRC peptides, the variable temperature CD data revealed a greater susceptibility to thermal disruption in the mixture containing HRN-S164P relative to the mixture containing the native HRN. These data indicate that HRN-S164P consistently forms less stable 6HB assemblies than does the native HRN.

### The destabilized F S164P can undergo activation and mediate fusion in the presence of the α/β-VI-8 peptide

**Fig 4:**
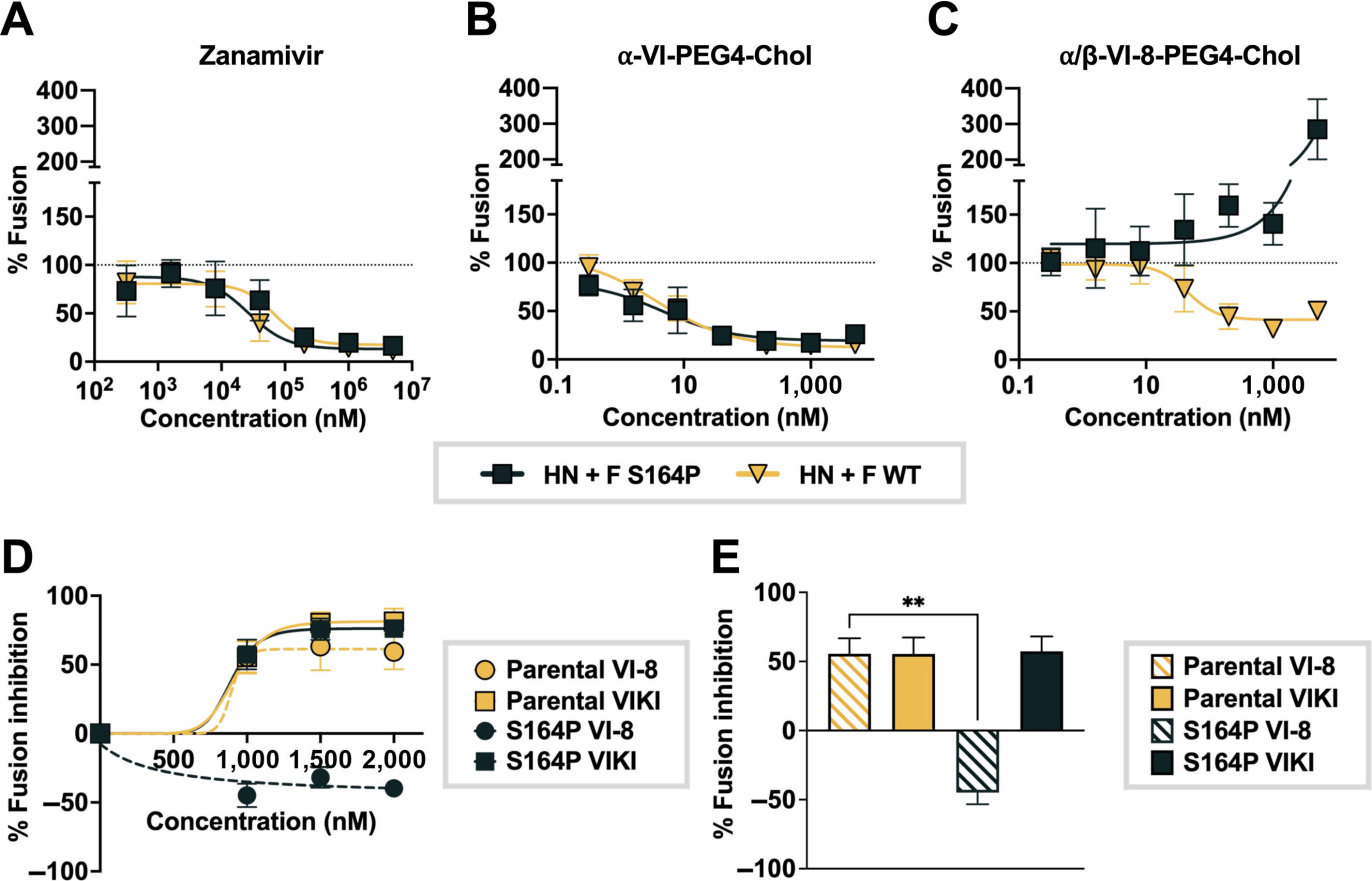
F S164P confers resistance to α/β-VI-8 in cell-cell fusion assay when paired with a highly fusogenic HN protein. (**A–C**) Cell-to-cell fusion mediated by HN + F was measured by beta-galactosidase complementation with cells expressing HN T193A and either the parental F or F S164P in the presence of (**A**) zanamivir, a compound that blocks HN receptor engagement and was previously shown to inhibit fusion at the range of concentrations shown on the *X*-axis; (**B**) α-VI-PEG4-Chol; and (**C**) α/β-VI-8-PEG4-Chol. Fusion values are normalized to the mean of untreated cells bearing respective HN/F complex in each experiment. Dotted line indicates 100% fusion, that is, no change in fusion relative to untreated cells. (**D**) Percent inhibition of fusion activation for F parental or F S164P in the presence of α-VIKI-PEG4-Chol or α/β-VI-8-PEG4-Chol. (**E**) Repeated measures one-way analysis of variance indicating significance in changes in fusion inhibition at 1,000 nM for (**D**). Data are means ± SE from three separate biological replicates for panels **A–E**.

When HPIV3 HN binds to a host cell surface receptor, it triggers the activation of the F protein (12, 13, 19–22). Prior to receptor engagement, HN maintains F in its pre-fusion state and prevents premature structural transitions in F (15, 48, 49). The efficiency of F-mediated fusion is regulated not only by F itself but also by the properties of HN including receptor avidity and F-triggering potency (15, 16, 44, 46, 50). More receptor-avid or more powerfully F-triggering HN proteins are more efficient at promoting fusion by the HN/F complex (16, 21, 44, 45). To assess the S164P F fusogenicity in the presence of α/β-VI-8-PEG4-Chol in the context of an HN-F complex that includes a highly fusion-promoting HN, we examined the effect of the HN bearing a T193A mutation in the sialic acid binding site (19, 51). We have previously shown that the T193A mutation in HN confers enhanced fusion promotion (45, 46, 51, 52). This residue comprises HN’s primary sialic acid binding site, the region of the HN protein that we have shown to be critical for activating HPIV3 F; alteration of this site affects fusion, infectivity, and viral fitness in vivo (16, 17, 44–47, 49). Using a cell-cell fusion assay where fusion of cells co-expressing HN/F pairs with receptor-bearing cells is quantitated by beta-galactosidase complementation, we determined the effect of this receptor-avid HN on inhibitor resistance mediated by S164P F compared to the parental F (Fig. 4). For comparison, we included the effect on fusion of zanamivir, a compound that blocks HPIV3 HN receptor engagement and thereby inhibits fusion (53) (Fig. 4A) and α-VI-PEG4-Chol (Fig. 4B), the all-α progenitor lipopeptide of α/β-VI-8-PEG4-Chol (Fig. 4C). Zanamivir and α-VI-PEG4-Chol inhibit the cell-cell fusion mediated by HN T193A/F S164P. However, with this fusion promoting HN, the F S164P confers resistance to α/β-VI-8-PEG4-Chol’s effect on F-mediated membrane fusion and, at high concentrations, leads to enhancement of fusion in the presence of α/β-VI-8-PEG4-Chol.

To further evaluate the impact of the F S164P mutation on the ability of F to be activated by HN and to complete fusion in the presence of inhibitory peptides, we used an assay that distinguishes between different states of F activation and quantitates each state (19, 54). The readout for F activation, insertion, and progress to fusion is fusion of red blood cells (RBCs) with F-expressing cells. Cells co-expressing F bearing S164P or parental F were bound to their sialic acid receptors on RBCs at 4°C and transferred to 32°C to permit F activation, in the presence of α/β-VI-8-PEG4-Chol or the all-α peptide, α-VIKI PEG4-Chol (Fig. 4D and E). In each condition, we measured the percentage of target RBCs that had undergone fusion. Fusion mediated by the parental virus F is similarly blocked by either peptide. Fusion mediated by the S164P F is inhibited by α-VIKI PEG4-Chol but not inhibited by α/β-VI-8-PEG4-Chol. We were surprised to observe that in this assay as well, the S164P F is more effective at completing fusion in the presence of the α/β-VI-8-PEG4-Chol peptide than in the absence of this peptide (black circles).

### HN contributes to peptide inhibitor sensitivity

To examine the effect of differences in HN’s triggering potency on F’s sensitivity to peptide inhibition, we assessed the effect of α-VI-PEG4-Chol and α-VIKI-PEG4-Chol on fusion mediated by parental F paired with either parental HN, the hyper-avid HN T193A (45, 46, 51, 52), or the hyper-triggering HN H552Q, a mutation that arises in field viruses forced to adapt to culture and confers more powerful activation of F (16, 44–47). In the presence of each of the more fusion-promoting HNs, significantly more peptide was required to achieve inhibition of F-mediated fusion (Fig. S3). For α-VI-PEG4-Chol, the inhibitory half maximal inhibitory concentration (IC_50_) was 20× higher in the presence of the HN H552Q with enhanced F-triggering (Fig. S3A and C), and similar increases were observed for α-VIKI-PEG4-Chol (Fig. S3B and C). The effect of the more efficiently fusion-promoting HNs is to increase the concentration of HRC peptide necessary to block the structural transition of F.

To determine whether similar differences in HN impact the function of the F bearing the destabilized 6HB, fusion of cells co-expressing HN/F paired with either the fusion-promoting lab-adapted (LA) HN bearing D556 (Fig. S3D), clinical isolate (CI-1) HN bearing N556 (Fig. S3E), or CI-1 HN bearing Q552 (Fig. S3F) and S164P F or parental F was quantitated by beta-galactosidase complementation. The LA HN is more efficient at activating F, compared to the CI-1 HN (16, 44, 47). For comparison, cells co-expressing F with influenza hemagglutinin (HA)—which binds sialic acid receptor but does not have F-triggering activity—were included in the fusion assay (Fig. S3G). In the presence of any HPIV3 receptor binding protein, the S164P F mediated more fusion than the parental F, and this difference was retained even with the highly fusion-promoting LA HN (Fig. S3D); the added triggering power does not compensate for the parental F’s lower fusion compared to the 6HB-destabilized F.

### Destabilizing the 6HB leads to a fitness cost in human airway

**Fig 5:**
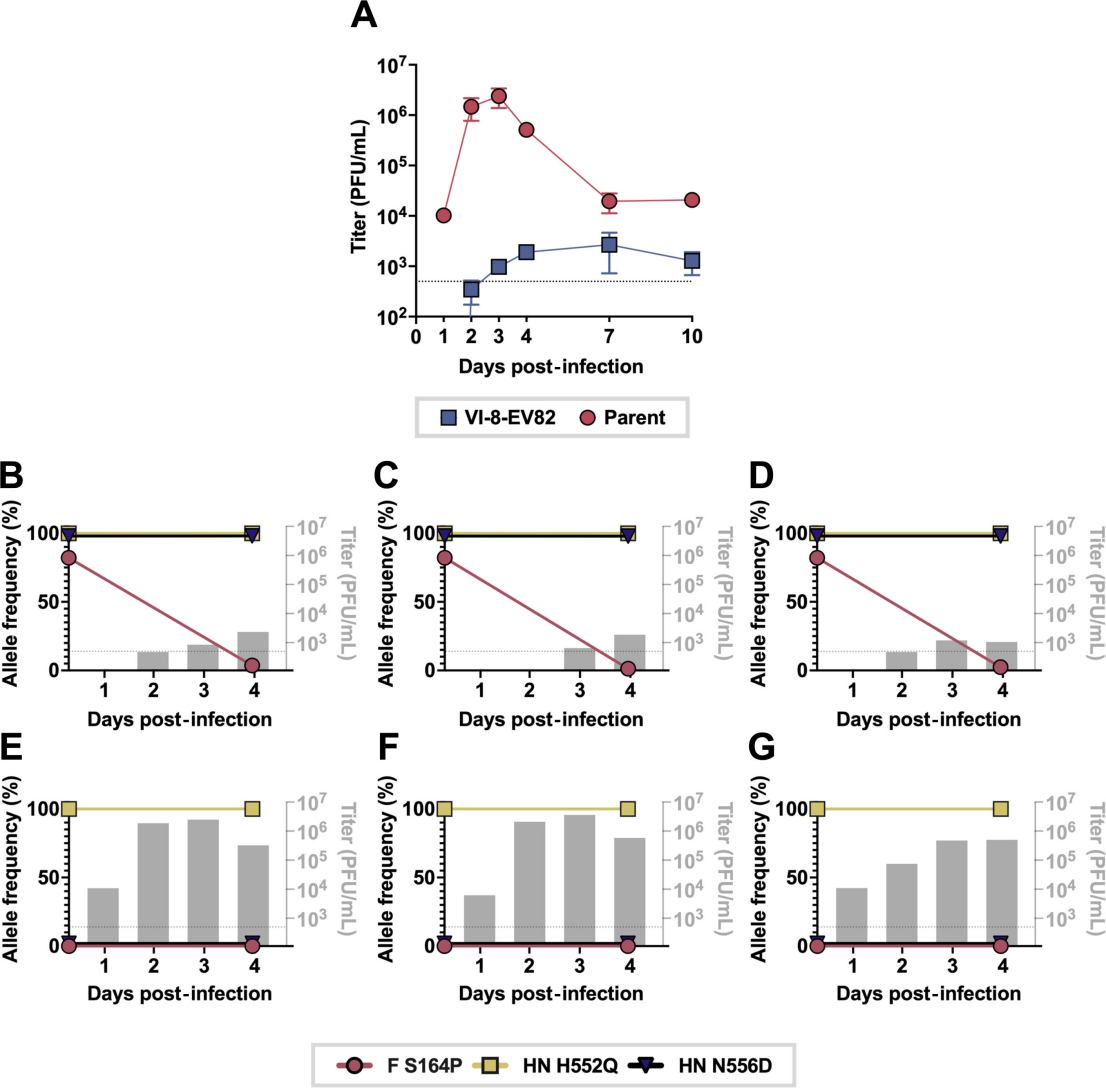
F S164P has reduced fitness in the airway. (**A**) Human airway epithelial (HAE) cells were infected with parental virus or VI-8-EV82 virus, and apical washes were titered daily for up to 10 days post infection. (**B–G**) Allele frequencies (points) of three HAE wells with either (**B–D**) VI-8-EV82 or (**E–G**) parental (CI-1 HN-H552Q) were measured at 0 and 4 days post infection. Titers were measured in plaque-forming units (PFU; gray bars) at 1, 2, 3, and 4 days post-infection. Dotted line indicates 500 PFU/mL detection limit for panels **A–G**. Data are means ± SE from three separate biological replicates for panel **A**.

To identify fitness consequences of the S164P F 6HB-destabilizing mutation paired with the D556 HN that emerged during selection with α/β-VI-8-PEG4-Chol, we evaluated infection with VI-8-EV82 (virus with 82% allele frequency of the F-S164P mutation) in human airway epithelia (HAE) (Fig. 5). These HAE cultures are an authentic model of the human lung, reflecting the natural tissue in terms of cell environment and selective pressure (47, 50), and have been validated for evaluating features of infection and HPIV3 fitness (50, 55, 56). We compared the growth and evolution of the VI-8-EV82 virus to the parental virus CI-1-HN H552Q. HAE cultures at an air-liquid interface were infected with resistant or parental viruses, and viral titer was measured daily for 10 days (Fig. 5A). Compared with the parental virus, the resistant virus showed significantly reduced production of infectious viral particles (blue line) which remained much lower than the parental level (red line) through day 10.

HN or F mutations that confer a disadvantage in the human airway generally lead to adaptation toward improved airway infection (44, 45, 57). The HN-F fusion complexes of HPIV3 clinical strains illustrate minimal evolution during growth in HAE (44, 47, 50, 55), and therefore, growing variant viruses in HAE can help determine the relative fitness cost of a given mutation as well as test for adaptation to HAE. Deep sequencing of the day 4 viruses collected from 3 HAE cultures for each virus (Fig. 5B through D) revealed that the allele frequency of the S164P mutation in F dropped from 82% to <5% in each sample, suggesting a significant fitness cost of this mutation in the airway. The S164P F was out-competed by the parental sequence under the selection pressure of growth in human airway, revealing the severe fitness cost of this change in the background of the field strain CI-1.

## DISCUSSION

The mechanism of HPIV entry involves fusion of the viral and cellular membranes, mediated by a precisely regulated interaction between the receptor-binding (HN) and fusion (F) proteins. This HN-F complex is finely tuned to the airway environment, ensuring efficient replication and spread (44, 47, 50, 55, 56). In the respiratory tract, strict control of F protein activation is essential to prevent excessive membrane fusion, which can impair infection. The refolding of the F protein, leading to the formation of a stable 6HB formed by the HRN and HRC, is central to the membrane fusion process (12, 13, 19–22). Here, we show that this step is sensitive to disruption by a mutation in the HRN that destabilizes the 6HB. The emergence of a virus with a structurally and functionally altered HN-F complex under the selection pressure of an HRC peptide highlights the capacity of the paramyxovirus fusion/entry complex to adapt to external stimuli that target highly conserved epitopes.

Our results demonstrate that HPIV3 can escape inhibition by a potent HRC peptide through the emergence of an F protein mutation, S164P, located in the HRN domain, alongside a previously characterized HN mutation, N556D, that decreases HN’s receptor-cleaving (neuraminidase) activity. The F mutation S164P destabilizes the six-helix bundle (6HB). Because 6HB stability is essential for membrane fusion, the S164P mutation creates a functional constraint that inflicts a significant fitness cost to evolving resistance. These findings highlight 6HB stability as a critical mechanistic barrier that fusion inhibitors exploit to restrict resistance.

The emergence of a resistance mutation in the HN dimer interface (N556D) was not surprising, since previous studies have shown that mutations in HN can dramatically influence fusion kinetics and viral infectivity (45, 47, 49). We have previously noted such mutations in HN that promote HN’s F-triggering function, enhance receptor avidity, or decrease receptor cleavage can assist entry in the presence of inhibitors (14, 28). HN molecules with a more effective triggering function achieved by any of these means promote enhanced kinetics of activation and may thus overcome the inhibitory effect of HRC peptides that interact strongly with the trimer HRN of F (14, 28, 36). HNs with powerful F-triggering can be envisioned as “pushing” the F past the impediment of an inhibitory peptide, providing strong enough activation to overcome an impaired F. It is the continued engagement of HN that subverts peptide inhibitory activity. The N556D mutation in HN’s dimer interface emerged not only during brief exposure to cell culture conditions (47) but also in persistently infected individuals (45), highlighting its consistent role in promoting enhanced fusion typically absent in circulating HPIV3.

The S164P mutation that emerged in the HRN domain of F causes a pronounced disruption in the α-helicity and stability of the 6HB, as revealed by circular dichroism and variable temperature CD experiments. This mutation was sufficient to confer resistance to α/β-VI-8-PEG4-Chol but did not affect susceptibility to the all-α peptides α-VIKI-PEG4-Chol or α-VI-PEG4-Chol. Along with 6HB destabilization, the F S164P mutant had increased capacity for fusion, in the presence of both a cell-culture-adapted HN protein bearing D556 and a clinical isolate HN bearing N556. We propose that the lower potency of the α/β-VI-8-PEG4-Chol relative to α-VI-PEG4-Chol, resulting from the lower affinity of α/β-VI-8 for the HPIV3 HRN, permitted the emergence of this F variant, aided by the enhanced-F-activating HN. This finding suggests the need for further optimization of the α/β-VI-8-PEG4-Chol prototype to maintain or improve resistance to proteolysis while also enhancing stability of the peptide-HRN 6HB.

Interestingly, similar proline substitutions to the HPIV3 HRN domain mutations found here have been used to destabilize the post-fusion conformations—and thereby stabilize the pre-fusion state—of several viral fusion proteins for the purpose of vaccine design or structural studies. For the soluble trimeric form of the envelope (env) glycoprotein complex of human immunodeficiency virus type 1 (“SOSIP”), proline substitutions in env were critical for stabilization (58–60), aiding the development of native pre-fusion conformations of the trimer that elicit broadly neutralizing antibodies. Similar pre-fusion conformation-stabilizing strategies have been key for both structural analysis and vaccine design for the respiratory syncytial virus fusion protein (61–63), resulting in the highly effective vaccine now in use (64, 65). For betacoronaviruses, the use of proline substitutions to stabilize the pre-fusion spike (S) protein was first worked out for Middle East respiratory syndrome and severe acute respiratory syndrome coronavirus (SARS-CoV) (66, 67) and later applied to SARS-CoV-2 (68–70), enabling detailed structural studies and effective vaccines. Parallel approaches are now being explored broadly for the design of paramyxovirus vaccines (71).

Fusion mediated by the S164P F was not only resistant to the inhibitory effect of α/β-VI-8-PEG4-Chol but also more effective in the presence of this peptide. One hypothesis to explain this finding lies in our demonstration that the HRN with S164P lost α-helicity relative to the native HRN, and that helical structure was regained in the presence of the α/β-VI-8-PEG4-Chol peptide. Thus, the apparent inhibitor dependence of this variant arose because the peptide restored the structure needed for membrane fusion, which had been compromised by the combined effects of the S164P HRN’s reduced helicity and its lowered affinity for its own HRC domain. The same effect would not be apparent with the all-α peptide because of its greater affinity for the HRN trimer. In other words, in the presence of S164P, the extended form of F can progress to the 6HB via displacement of the α/β-peptide, but the higher-affinity all-α peptide cannot be displaced. The dependence of the mutated virus on the presence of the α/β-VI-8-PEG4-Chol peptide is similar to the observation of HIV-1 variants that emerged during treatment with the peptide fusion inhibitor T20 (enfuvirtide, fuzeon) (72). T20 escape variants bear HRN mutations that have a destabilizing effect on the post-fusion six-helix bundle, similar to the S164P variant (72, 73). Single mutations in both the HRN and HRC domains in one patient led to a hyperfusogenic variant that was dependent on the antiviral peptide for replication, apparently because the peptide partially prevented premature conformational transition to the post-fusion state and resultant inactive virions (72). This phenomenon was dose-dependent; high T20 levels completely blocked the transition and blocked entry.

Despite the S164P mutated F protein’s ability to mediate fusion under inhibitory pressure, the mutation exacts a significant fitness cost in a physiologically relevant human airway epithelial (HAE) model. This finding is consistent with our previous observations that enhanced fusogenicity of the HN-F complex, whether conferred by alterations in HN or F, decreases fitness in human airway (47, 49, 56). The rapid loss of the S164P allele frequency during viral replication in HAE strongly suggests that the destabilized 6HB impairs viral fitness *ex vivo* and would impair fitness *in vivo*. This observation is in contrast to S164P allele enrichment in peptide-containing *in vitro* culture, with a dynamic positive selection in the presence of the α/β-VI-8-PEG4-Chol and negative selection for viral spread in the absence of the antiviral peptide. This suggests a fitness disadvantage for the S164P mutated F protein and a tailoring of F-S164P allele frequency to match the constraints of the current environment. This observation highlights an existential trade-off where the escape variants are not only resistant but dependent on the treatment to persist and consequently are unfit to spread and replicate in naive untreated host environments.

## MATERIALS AND METHODS

### Virus growth and purification

Recombinant viruses were generated by reverse genetics using an HPIV3 clinical isolate one sequence (50) containing an mCherry cassette between genes P and M. Resulting viruses were propagated using Vero cells (American Type Culture Collection [ATCC]), Calu-3 cells (ATCC), or HAE EpiAirway AIR-100 cultures (MatTek Corporation). Viruses were titered by limiting dilution infection of Vero, and infected cells were quantified using a Cytation 5 imager (Biotek). All recombinant viruses were sequenced using metagenomic next-generation sequencing (mNGS) prior to experimental use (74).

### Constructs

Plasmids encoding HPIV3 HN and F were generated through site-directed mutagenesis of a previously constructed pCAGGS mammalian expression vector (57). The sequence of the JS strain of HPIV3 was acquired from the NIH (Z11575.1). Consensus sequence of the laboratory-adapted strain of HPIV3 (Wash/47885/57) used throughout the study was obtained from the NIH (HA-1, NIH no. 47885, catalog no. V323-002-020) (75).

### Cells

HEK293T cells (ATCC; CRL-3216) for transfections were grown in Dulbecco’s modified Eagle medium (DMEM) (Gibco) supplemented with 10% fetal bovine serum (FBS) and 1% penicillin-streptomycin (Gibco) at 37°C and 5% CO_2_. Vero cells (ATCC; CCL-81) were grown in DMEM supplemented with 10% FBS and 1% penicillin-streptomycin at 37°C and 5% CO_2_. Calu-3 lung adenocarcinoma (ATCC; HTB-55) cells were grown in Eagle’s minimum essential medium (ATCC) supplemented with 10% FBS and 1% penicillin-streptomycin at 37°C in 5% CO2. All cells tested negative for mycoplasma presence using MycoStrip Mycoplasma Detection Kit per the manufacturer’s specifications (Invivogen).

### Chemicals and peptides

Zanamivir (Acme Bioscience) was dissolved in Opti-MEM at a concentration of 50 mM and stored at −20°C. α-VI-PEG4-chol, α-VIKI-PEG4-chol, and α/β-VI-8-PEG4-chol fusion inhibitory peptides were produced by standard Fmoc-solid phase methods, and the cholesterol moiety was attached using displacement of an α-bromoamide (50). Bromoacetyl-PEG4-cholesterol was custom synthesized by Charnwood Molecular (UK). α-VI-PEG4-chol, α-VIKI-PEG4-chol, and α/β-VI-8-PEG4-chol (5 mM in DMSO) were kept at −80°C.

### Viral selection strategy

Vero cells (ATCC) in a 96-well plate were infected with 200 plaque-forming units (PFUs) per well of CI-1-HN H552Q (50) in Opti-MEM (Gibco) supplemented with 1% penicillin-streptomycin. After 2 h, the infection medium was replaced with decreasing concentrations of α-VIKI-PEG4-chol or α/β-VI-8-PEG4-chol peptide (2,000 nM; 1:2 dilutions down to 7.8 nM). Viral spread was observed in wells treated with 2,000 nM α/β-VI-8-PEG4-chol. Six days post-infection, viruses that spread in the presence of lipopeptide and untreated control viruses were sequenced. Additional aliquots of virus were collected and frozen at −80°C.

### Viral entry inhibition assay

Vero cells (ATCC) in a 96-well plate were infected with 200 PFUs parental (CI-1 HN H552Q) or VI-8-EV36 virus in the presence of α-VIKI-PEG4-chol, α-VI-PEG4-chol, or α/β-VI-8-PEG4-chol and incubated for 2 h at 37°C. Following this incubation, cells were overlaid with 0.5% carboxymethyl cellulose (17). After 24 h, cells were imaged on a Cytation 5 imager (Biotek), and fluorescently labeled cells were counted. Inhibition of entry was quantified by comparing the number of infected cells at different concentrations of peptide to the number of infected cells in the absence of peptide for each virus.

### Plaquing variant viruses

Vero cells (ATCC) in a six-well plate were infected with 200 PFUs of VI-8-EV36 virus for 2 h at 37°C. Following infection, cells were overlaid with 1:2 dilutions of α/β-VI-8-PEG4-chol with a maximal concentration of 2,000 nM 0.5% carboxymethyl cellulose. Cells were imaged daily on a Cytation 5 imager (Biotek), and total fluorescence intensity was recorded. All viruses were collected at 6 dpi and sequenced using mNGS.

### β-Gal complementation-based fusion assay to assess fusion promotion by HN/F

We previously adapted a fusion assay based on α complementation of β-galactosidase (19, 76). In this assay, receptor-bearing cells expressing the Ω peptide of β-Gal were mixed with cells co-expressing designated envelope glycoproteins and the α peptide of β-Gal, and cell fusion leads to complementation. Fusion was stopped by lysing the cells. Substrate was added (Galacton-Star substrate; Applied Biosystems), and luminescence was read after 1 h at 1,000 ms on a Tecan M1000 Pro. Relative IC_50_ values were determined by fitting points with a four-parameter, least squares regression.

### Measurement of F-activation and fusion between RBCs and envelope glycoprotein-expressing cells

HEK 293T cells were transiently transfected with HPIV3 HN D216R and either HPIV3 F or HPIV3 F S164P, for 3–5 h at 37°C. Cells were then treated overnight with 15 mU/well of exogenous neuraminidase (Sigma Aldrich) to deplete sialic acid receptors. Cells were then washed and incubated with 1% RBC suspensions for 30 min at 4°C. After the samples were washed to remove unbound RBCs, they were treated with 1:2 dilutions of 2 µM α-VIKI-PEG4-chol, and α/β-VI-8-PEG4-chol and brought to 32°C for 60 min. The plates were rocked, and the liquid phase collected in V-bottom plates for measurement of released RBCs. Next, 10 mM zanamivir was added to collect reversibly bound RBCs. V-bottom plates were spun down, and pelleted RBCs were lysed in milli-Q water and transferred to a flat bottom 96-well plate for quantitation. The cells were then incubated with RBC lysis solution (ammonium-chloride-potassium lysis buffer; Thermo Fisher), where the lysis of unfused RBCs removes unfused cells. The liquid phase was collected in flat-bottom plates for measurement of irreversibly bound RBCs. The cells were then lysed in 10% dodecyl maltoside HEPES (DH) buffer (5 mM HEPES, 10 mM NaCl, and dodecyl maltoside 0.5 mg/mL]) 1:10 in phosphate-buffered saline (PBS) and transferred to flat-bottom 96-well plates for quantification of fused RBCs. Hemoglobin absorbance for the above three compartments was determined by measuring absorbance at 405 nm on a Tecan M1000 Pro.

### Viral infection of HAE

The HAE EpiAirway AIR-100 system (MatTek Corporation) contains cultured human-derived tracheo/bronchial epithelium that forms pseudostratified, differentiated mucociliary epithelium recapitulating *in vivo* human tissue (55). Upon receipt from the manufacturer, HAE cultures were transferred to provided six-well plates containing 1 mL of AIR-100-ASY assay medium (MatTek Corporation) per well with the apical surface remaining exposed to air and incubated at 37°C in 5% CO2 overnight prior to infection. HAE cultures were infected with 10,000 PFU per well of CI-1 HN H552Q or VI-8-EV82 virus at the apical surface for 3 h at 37°C, followed by inoculum removal. Maintenance medium (MatTek Corporation) was changed every other day with 1 mL medium via the basolateral surface. Viruses were harvested by adding 200 μL of provided 1× PBS containing magnesium and calcium per well via the apical surface and incubated for 30 min at 37°C. Supernatant was subsequently collected and viral titers were determined by limiting dilution infection of Vero cells (ATCC), with infected cells quantified using Cytation 5 (BioTek). When quantifying titer, a detection limit of 500 PFU/mL was set. All viral genomes were sequenced.

### Sequencing library generation and analysis

Due to low viral load, viral whole-genome sequencing (WGS) for “Viral Infection of HAE” samples was performed using the QIAseq xHYB Microbial Hyb&Lib Kit A (Qiagen Cat No. 334525) and a QIAseq xHYB custom probe panel (Qiagen Cat No. 334565) per the manufacturer’s protocol. Briefly, RNA was converted to double-stranded cDNA, enzymatically fragmented, end-repaired, indexed, purified (via 0.9× and 1.1× QIAseq bead cleanups), and PCR amplified (14 cycles) before final bead purification at a 1.1× QIAseq bead ratio. Pre-capture libraries were pooled based on the viral titer values (PFU/mL; 4 samples per pool), dried down, resuspended, and hybridized overnight with the custom biotinylated probe panel containing HPIV3 oligos. Probe-bound targets were captured using streptavidin-coated magnetic beads, washed (to remove non-specific fragments), PCR-amplified (20 cycles), and purified using a 1.1× bead cleanup. Finally, library concentrations were measured with the Qubit 4 Fluorometer and Qubit 1× dsDNA HS Assay Kit (ThermoFisher, Q33230). Library size was verified using Agilent Tapestation 4200 on D1000 Screentapes (Agilent, 5067-5582). Libraries were sequenced on Illumina NextSeq 2000 instruments using a 1 × 100 bp read format.

For the rest of the HPIV3 samples, WGS was carried out using a previously described metagenomic next-generation sequencing approach (74). Libraries were sequenced on either Illumina NextSeq 2000 or NovaSeq 6000 platform with 2 × 150 bp or 1 ×1 00 reads.

### Data analysis

Raw reads were trimmed and quality filtered with fastp (v0.23.4) (77). Filtered reads were then used for variant calling using the RAVA workflow (default parameters) and the respective plasmid map sequence as a reference (https://github.com/greninger-lab/RAVA_Pipeline/tree/2025-08-05_HPIV3_Crosby_et_al) (78, 79).

### Circular dichroism

All circular dichroism (CD) experiments were performed on a JASCO J-1500 CD spectrometer. Samples were prepared in 1 mm quartz cuvettes with 10 mM phosphate buffer, pH 7.4. Wavelength scans were collected from 190 to 260 nm with a 1 nm bandwidth, 4 s averaging time, and scanning speed of 50 nm/min. Individual peptides were prepared at 25 µM, and peptide combinations were prepared at 100 µM (50 µM for each component). For variable temperature experiments, ellipticity was measured at 222 nm as temperature was raised from 5°C to 95°C in 5°C increments with a 5 min equilibration time at each new temperature and a 5 s averaging time for each measurement.

### Statistics

Graphs were generated and statistical analysis was performed with GraphPad Prism 10. Results are means ± SEM unless otherwise stated. *P*-values less than 0.05 were considered statistically significant.

### Reverse transcription and qPCR

To measure the amount of viral genome released, 140 µL of fluid containing released virus was processed for RNA extraction with the QIAamp Viral RNA Mini Kit (Qiagen), reverse transcribed (High-Capacity cDNA Reverse Transcription Kit; Thermo Fisher), and quantified by quantitative PCR (qPCR) with HPIV3-specific primers relative to a Comprehensive Microbiota Control v2.0 (Thermo Fisher).

## Data Availability

Raw data for figures and plasmid sequences encoding HN, HA, and F have been deposited in the Dryad repository (https://doi.org/10.5061/dryad.wh70rxx1d). Sequencing reads are available in NCBI BioProject PRJNA1302008. All other relevant data are within the paper and its supplemental material. Materials are available by MTA with the Trustees of Columbia University, New York. Reagents are available from the corresponding authors under a material agreement with Columbia University.

## SUPPLEMENTAL MATERIAL

Supplemental figures mbio.02741-25-s0001.docx Fig. S1 to S3.

ASM does not own the copyrights to Supplemental Material that may be linked to, or accessed through, an article. The authors have granted ASM a non-exclusive, world-wide license to publish the Supplemental Material files. Please contact the corresponding author directly for reuse.
