# Supplementary material for: A fusion protein’s weak link: functional constraints revealed by inhibitory peptide interaction with the parainfluenza fusion protein
Source: mBio. 2026 Apr 14;17(5):e02741-25. doi: 10.1128/mbio.02741-25 (PMC13170234; doi:10.1128/mbio.02741-25)
Supplement: Supplemental figures — Fig. S1 to S3. [file mbio.02741-25-s0001.docx]

# **Supplemental figures:**

**Supplemental figure 1: S164P allele frequency and fitness in the absence of α/β-VI-8-PEG4-Chol.**

**(A)** VI-8-EV36 was passaged in Vero cells and was harvested and sequenced at 3 dpi, yielding “VI-8-EV10” with virus bearing F S164P at 10% allele frequency. **(B)** PFU/genome copy was calculated by normalizing the infectious titer of virus bearing F S164P at 95% allele frequency (VI-8-EV95) and parent virus (in the presence and absence of 100 nM α/β-VI-8-PEG4-Chol) to the number of HPIV3 genome copies. A two-way ANOVA and multiple comparison tests were performed to determine the significance of changes in PFU/genome copy relative to untreated VI-8-EV95 for **(B)**. Mean and standard deviation in PFU/genome copy are shown for **(B)** across three technical replicates.

**Supplemental figure 2: HRN-S164P forms a helical structure at high concentration.**

Full-spectrum circular dichroism scan at 25°C of HRN S164P peptides at 50, 100, 200, or 300 µM.

**Supplemental figure 3: HN influences F’s peptide resistance and modulates the fusion by the destabilized F S164P.**

Cells expressing F and the wild-type HN, HN-H552Q, or HN-T193A were treated with **(A)** α-VI-PEG4-Chol or **(B)** α-VIKI-PEG4-Chol to examine differences in HN/F complex susceptibility to lipopeptide. Fusion values were normalized to the mean of respective LA HN + F complexes for each experiment. Relative IC50 values are indicated **(C)**, with α-VI-PEG4-Chol ("VI") and α-VIKI-PEG4-Chol ("VIKI"). **(D-G)** Cell-to-cell fusion was measured by beta-galactosidase complementation with cells expressing **(D)** LA HN, **(E)** CI-1 HN, **(F)** CI-1 HN H552Q and **(G)** HA HN paired with F S164P (blue) or F parental (red). Relative fusion was normalized to the mean of the indicated HN with F-parental for each experiment to determine fold change (FC) relative to the parental HN/F. Data for **(D-G)** are means ± SE from five separate biological replicates. Statistically significant differences between mean values were determined using an unpaired two-tailed t test.
